# Number and distribution of nutrient foramina within the femoral neck and their relationship to the retinacula of Weitbrecht: an anatomical study

**DOI:** 10.1007/s12565-015-0319-5

**Published:** 2015-12-21

**Authors:** Jiong Mei, Ming Ni, Guoliang Wang, Guangyao Jia, Shiwei Liu, Xueliang Cui, Chao Jiang, Hua Wang, Yahui Dai, Kun Quan, Rui Chen

**Affiliations:** 1 Department of Orthopaedics, Tongji Hospital, Tongji University School of Medicine, 389 Rd. Xincun, Shanghai, 200065 China; 2 Department of Medical Imaging, Tongji Hospital, Tongji University School of Medicine, Shanghai, 200065 China

**Keywords:** Avascular necrosis, Femoral neck, Nutrient foramina, Vascular anatomy, Retinacula of Weitbrecht

## Abstract

Precise knowledge of the vascular supply of the femoral head is critical when contemplating surgery around the femoral head and neck junction. To determine the blood supply to the femoral neck, 2417 nutrient foramina from 76 cadaveric specimens were analyzed based on size, number, and distribution. Within the subcapital, transcervical, and basicervical regions of the femoral neck, the largest numbers of nutrient foramina were found on the superior (lateral) surface, followed by the anterior and posterior surfaces, and then the inferior (medial) surface (all *p* < 0.001). The diameters of most of the nutrient foramina were less than 1 mm. For the posterior and superior surfaces, the nutrient foramina in the basicervical region were significantly larger than those within the transcervical or subcapital regions (nutrient foramina >2 mm posteriorly: 23.6, 12.7, and 9.0 % in the basicervical, transcervical, and subcapital regions, respectively; superiorly: 23.7 vs. 15.4 vs. 16.8 %, respectively). In conclusion, neither the anterior nor the inferior surfaces in the basicervical, transcervical, and subcapital regions showed any significant differences in nutrient foraminal size. The areas containing densely distributed nutrient foramina were consistent with the regions covered by the retinacula of Weitbrecht.

## Introduction

Precise knowledge of the vascular supply to the femoral head is critical when contemplating surgery in the region surrounding the femoral head and neck junction (Lavigne et al. 2005). The blood supply to the femoral head is derived primarily from the medial femoral circumflex artery (MFCA) and the lateral femoral circumflex arteries (LFCAs) (Trueta and Harrison 1953). The LFCA supplies the majority of the inferoanterior femoral head with blood, but the largest contributor to the blood supply of the femoral head is the MFCA, which supplies its superolateral aspect (Trueta and Harrison 1953). In femoral neck fractures, the lateral ascending cervical branches of the MFCA are at risk of disruption. Loss of this blood supply increases the risk for avascular necrosis (AVN) of the femoral head (Canale 1998).

Both the MFCA and LFCA give off branches from the joint capsule at the base of the femoral neck when passing through the retinacula of Weitbrecht to the femoral head and neck (Gojda and Bartonicek 2012). Thus, the retinacula of Weitbrecht can be considered a bridge for blood vessels entering the femoral head. Disruption or distortion of these retinacular branches to the femoral head due to fracture displacement plays a significant role in the development of osteonecrosis (Arnoldi and Lemperg 1977).

It has been confirmed by evidence-based medicine that the incidence of AVN of the femoral head is related to the degree of fracture displacement and the quality of diaplasis (Wright 2009; Lu-Yao et al. 1994). Toh et al. (2004) reported that the incidence of AVN of the femoral head was 11 %. Adverse prognostic factors included age, degree of fracture displacement, and state of fracture diaplasis (Barnes et al. 1976). Therefore, it is critical to determine the degree of injury to the blood supply to the femoral head after femoral neck fracture and retain as much of the residual blood supply to the femoral head as possible during treatment.

Though current studies regarding the blood supply to the proximal femur provide a rough distribution of the blood supply to the femoral head and neck (Gojda and Bartonicek 2012), most studies do not take into consideration the retinacula of Weitbrecht (Gojda and Bartonicek 2012).

We hypothesized that the areas of the femoral neck containing the most densely distributed nutrient foramina lay within the retinacula of Weitbrecht. Since there is likely to be a correlation between severity of injury to the retinacula and subsequent development of AVN, such a finding has significant clinical repercussions.

## Materials and methods

Our study protocol was approved by our Institutional Review Board.

A total of 76 dry specimens of adult femora were obtained from the Department of Anatomy, Tongji University School of Medicine. General information regarding these specimens, such as age, gender, presence of hip disease, and cause of death, was unknown.

Specimens were selected according to the following criteria: (1) no significant osteoarthritis or morphological changes within the femoral head; (2) the epiphyseal growth plate of the femoral head was closed; (3) the femoral neck was intact.

**Fig. 1:**
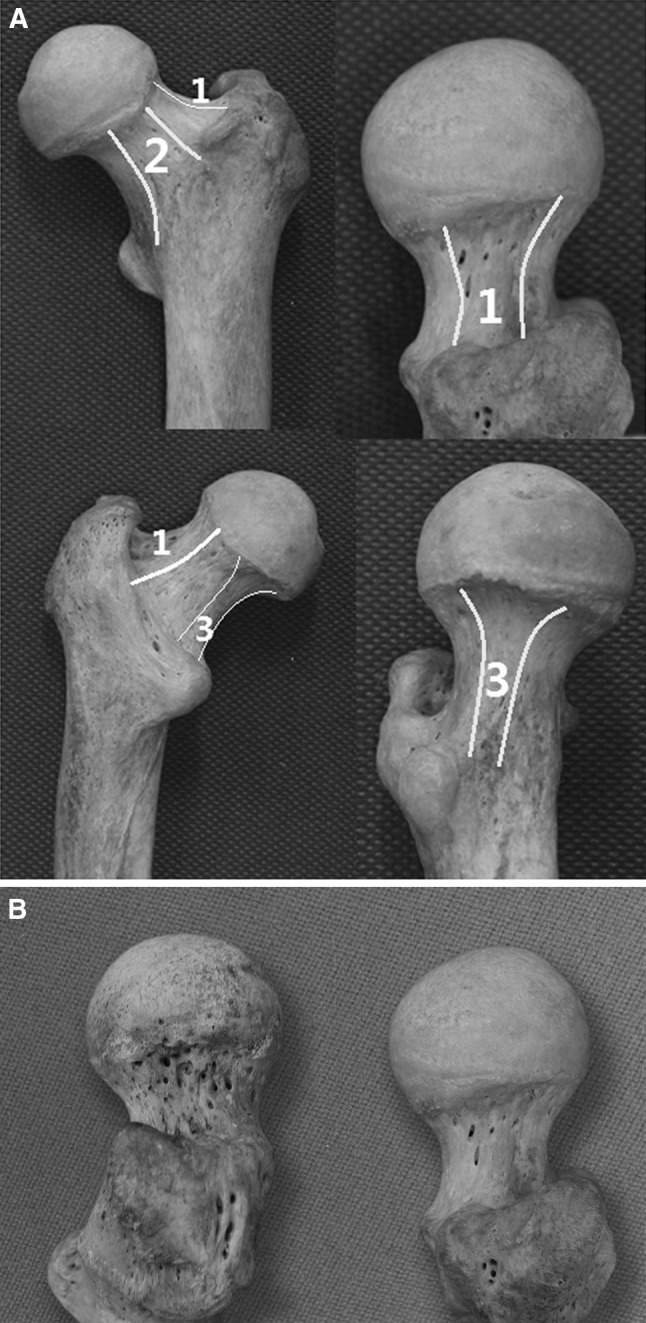
**a** Illustration of femoral neck surfaces in relation to the retinacula of Weitbrecht. *1* The region covered by the upper (lateral) retinacula. *2* The region covered by the anterior retinacula. *3* The region covered by the lower (medial) retinacula. **b** Individual differences between specimens. In most cases, the nutrient foramina were densely distributed in the region covered by the upper (lateral) retinacular band, but the nutrient foramina were sparsely distributed in a few cases, and this type of individual difference could also be observed in the region covered by the anterior or lower (medial) retinacular bands

Femoral neck surfaces in relation to the upper (lateral), anterior, and lower (medial) retinacula of Weitbrecht are illustrated in Fig. 1a. The retinacula of Weitbrecht were located on the femoral neck based on data provided by related papers (Gojda and Bartoníček 2012; Noriyasu et al. 1993; Bartonícek 1990; Guli et al. 2012) together with data obtained from our own observations of the retinacula of Weitbrecht in fresh and fixed specimens during our anatomical studies.

The basal (basicervical), middle (transcervical), and subcapital regions of the femoral neck were observed macroscopically, as previously described (Davidovitch et al. 2010). Delineation of the subcapital, middle (transcervical), and basal (basicervical) areas of the femoral neck was performed as described by Lavigne et al. (2005). Photographs of the superior, inferior, anterior, and posterior aspects of the femoral neck were taken. A line was then drawn along the margin of the articular surface of the femoral head in each aspect, and a parallel line was then drawn 1 cm distal to the first line. The region between these two lines was named the subcapital area. A line was then drawn along the junction between the femoral neck and the greater and lesser trochanters, and two parallel lines were then drawn 0.5 cm proximal and distal, respectively, to this line. The region between the latter two lines was defined as the basal (or basicervical) area. The region between the subcapital area and the basal area was then named the middle (or transcervical) area.

### Measurement of foraminal size and number

The blunt ends of Kirschner wires (K-wires) (Smith and Nephew, Germany) were used for the size measurements. Two wire diameters were used in the present study, 1.0 mm dia. × 150 mm and 2.0 mm dia. × 150 mm. Since most of the foramina had irregular round openings, the minimum diameter of each foramen was measured and that measurement was taken as its standard diameter. We oriented each specimen vertically (Fig. 1b) and used the vertical surface of the femoral neck to measure the foraminal size and the number of foramina. Our goal was not to obtain the precise number of nutrient foramina but to verify that there were significant differences between various individuals in the size and number of nutrient foramina within the femoral neck.

Foramina within the same bone specimen were counted using a computer. Each measurement (size or number) was carried out by two observers. If the results differed between the two observers, a consensus was reached after additional review.

### CT scanning of the proximal femora

CT scans of six proximal femora with obvious foramina were performed using a multidetector CT (Toshiba Aquilion ONE 320-slice spiral CT). The scanning parameters were 80 kV, 200 mA, a slice thickness of 0.5 mm, and a tube rotation of 0.35 s. All data were transferred to a workstation for postprocessing. Techniques used to obtain multiplanar three-dimensional reconstruction of images included three-dimensional surface rendering, maximum intensity projection (MIP), shaded surface display, volume rendering (VR), and multiplanar reformation (MPR). The surface of each proximal femur was reconstructed and the differences between the reconstructed femur and the specimen were analyzed.

### Statistical analysis

Data from the 76 specimens were expressed as counts and percentages except for the size of the nutrient foramina at various locations within the femoral neck. Fisher’s exact test was performed to evaluate the association between nutrient foraminal size and foraminal location on the femoral neck. Regarding the number of nutrient foramina within the 76 specimens, data were expressed as the median (interquartile range, the range from the 1st to the 3rd quartile) and tested by the Friedman test. When a significant result was obtained, the differences between the two locations on the femoral neck were then examined by a Wilcoxon signed rank test for post hoc analysis. The statistical assessments were two-sided and evaluated with a significance level of 0.05. When post hoc tests were necessary, a significance level of 0.01 was applied. All analyses were performed using the SPSS 15.0 statistical software package (SPSS Inc., Chicago, IL, USA).

## Results

### Numbers of nutrient foramina on the various surfaces

Among the 76 specimens, the number of nutrient foramina observed on the posterior and superior (lateral) surfaces of the basicervical femoral neck were significantly greater than the number observed on anterior or inferior (medial) surfaces (all *p* < 0.001). In the subcapital region of the femoral neck, more nutrient foramina were observed on the superior (lateral) surface when compared with other surfaces (all *p* ≤ 0.001). In the transcervical region of the femoral neck, the largest numbers of nutrient foramina were found on the superior (lateral) surface, followed by the anterior and posterior surfaces and the inferior (medial) surface (all *p* < 0.001) (Table 1).

**Table 1:** Distribution of nutrient foramina at various locations on the femoral neck in 76 cadaveric specimens

	Number of nutrient foramina (*n* = 76)			
	Anterior	Posterior	Superior (lateral)	Inferior (medial)
Basicervical	1.5 (1, 2)	3 (3, 5)^a^	4 (3, 5)^a^	0 (0, 2)^b,c^
Subcapital	1 (0, 3)	2 (0, 4)^d^	6.5 (5, 8)^a,b,d^	0 (0, 2)^b,c^
Transcervical	2 (1, 3)	1 (0, 3)^d^	6 (4, 8)^a,b,d^	0 (0, 0)^a,b,c,d,e^

Data are expressed as median (interquartile range, i.e., the range from the 1st to the 3rd quartile)

Subcapital: below the femoral head, transcervical: across the mid-femoral neck, basicervical: across the base of the femoral neck

^a^Indicates a significant difference when compared to anterior

^b^Indicates a significant difference when compared to posterior

^c^Indicates a significant difference when compared to superior

^d^Indicates a significant difference when compared to basicervical

^e^Indicates a significant difference when compared to subcapital

No significant differences in the number of nutrient foramina were observed between the basicervical, subcapital, and transcervical regions on the anterior femoral neck surface. On the posterior femoral neck surface, however, the number of foramina was significantly higher within the basicervical region than within the subcapital (*p* < 0.001) or transcervical regions (*p* = 0.002). Conversely, on the superior (lateral) femoral neck surface, fewer nutrient foramina were found within the basicervical neck region than within the subcapital and transcervical regions (both *p* < 0.001). On the inferior (medial) femoral neck surface, a significant reduction in the number of foramina was observed in the transcervical region compared with the basicervical and subcapital regions (both *p* < 0.001) (Table 1).

### Nutrient foraminal size differences among various regions

The sizes of a total of 2417 nutrient foramina from the 76 specimens were measured. The diameters of most of the nutrient foramina were less than 1 mm, although several ranged between 1 and 2 mm and a few were >2 mm.

For the anterior and inferior (medial) surfaces of the femoral neck, no significant differences in nutrient foraminal size were observed among the basicervical, transcervical, or subcapital regions. For the posterior and superior (lateral) surfaces, nutrient foraminal size in the basicervical region was significantly larger than those within the transcervical and subcapital regions (nutrient foramina >2 mm posteriorly: 23.6, 12.7, 9.0 % in the basicervical, transcervical, and subcapital regions, respectively; nutrient foramina >2 mm superiorly: 23.7, 15.4, 16.8 %, in the basicervical, transcervical, and subcapital regions, respectively) (Table 2).

**Table 2:** Size distribution of nutrient foramina in various femoral neck locations

	Total number of nutrient foramina			*p* value
	Basicervical	Transcervical	Subcapital	
Anterior (mm)				
<1	81 (66.9 %)	91 (65.5 %)	98 (75.4 %)	0.417
1–2	25 (20.7 %)	33 (23.7 %)	21 (16.2 %)	
>2	15 (12.4 %)	15 (10.8 %)	11 (8.5 %)	
Posterior (mm)				
<1	147 (53.3 %)	87 (69.0 %)	138 (73.0 %)	<0.001*
1–2	64 (23.2 %)	23 (18.3 %)	34 (18.0 %)	
>2	65 (23.6 %)	16 (12.7 %)	17 (9.0 %)	
Superior (lateral) (mm)				
<1	158 (52.0 %)	247 (55.9 %)	252 (51.0 %)	0.015*
1–2	74 (24.3 %)	127 (28.7 %)	159 (32.2 %)	
>2	72 (23.7 %)	68 (15.4 %)	83 (16.8 %)	
Inferior (medial) (mm)				
<1	60 (69.0 %)	13 (68.4 %)	72 (80.0 %)	0.066
1–2	14 (16.1 %)	6 (31.6 %)	12 (13.3 %)	
>2	13 (14.9 %)	0 (0.0 %)	6 (6.7 %)	

Data are expressed as count and percentage

* Indicates a significant association was observed

### Morphology and distribution of nutrient foramina within the retinacula of Weitbrecht

Nutrient foramina with different shapes were observed throughout the femoral neck. These foramina were generally round or oval. Several bony grooves could be observed in some cases. These grooves were formed by the connection of two or more relatively large foramina. The foramina burrowed in irregular directions within the femoral neck: some were vertical to the bone surface, while others were inclined obliquely in varying directions. The areas containing densely distributed nutrient foramina were consistent with the regions covered by the upper (lateral), anterior, and lower (medial) bands of the retinacula of Weitbrecht (Gojda and Bartonicek 2012) (Fig. 1).

Most nutrient foramina outside the area covered by the retinacula of Weitbrecht formed the entrances for the communicating branches between the different bands, and others were formed by blood vessels originating above, from the MFCA and LFCA at the base of the femoral neck.

There were obvious individual differences between specimens. In most cases, the nutrient foramina were densely distributed in the region covered by the upper (lateral) retinacular band, but the nutrient foramina were sparsely distributed in a few cases, and this kind of individual difference was also observed in the region covered by the anterior or lower (medial) retinacular bands (Fig. 1).

### Comparison between the CT images and the dry bone specimens

**Fig. 2:**
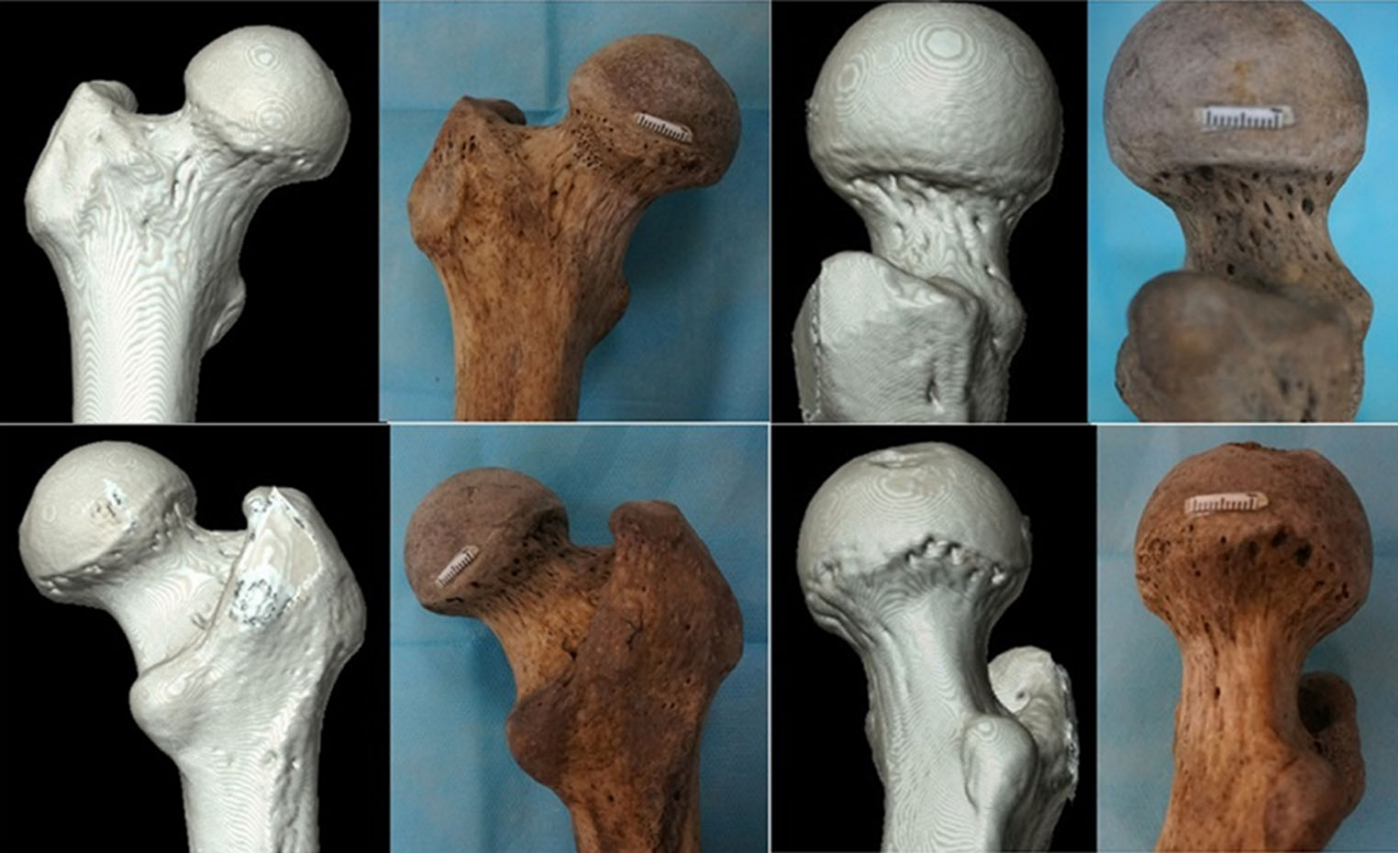
Comparison between images reconstructed from CT (*left*) and the dry bone specimens (*right*). Even an advanced CT scanning system could not delineate the relatively small nutrient foramina

CT scanning with 3D reconstruction was unable to delineate nutrient foramina, at even the maximum resolution of the images (Fig. 2).

## Discussion

We found that the largest numbers of nutrient foramina occurred on the superior (lateral) surface, followed by the anterior and posterior surfaces and the inferior (medial) surface of the femora (all *p* < 0.001). The diameters of most of the nutrient foramina were less than 1 mm. For the posterior and superior surfaces, nutrient foraminal size in the basicervical region was significantly larger than those in the transcervical or subcapital regions. No significant differences in nutrient foraminal size among regions were observed for either the anterior or the inferior surface. The areas containing densely distributed nutrient foramina were consistent with the regions covered by the retinacula of Weitbrecht, and therein lies the study’s novelty.

A number of studies have been published concerning the nutrient foramina within the femur. Most studies evaluating numbers, locations, and levels of femoral nutrient foramina (Santolini et al. 2014; Imre et al. 2012; Murlimanju et al. 2011a, b, c) have focused primarily on the shaft or diaphysis of the femur; few have been based on the MDCT (Imre et al. 2012). None of those studies provided conclusive evidence concerning the number of foramina (Santolini et al. 2014).

Our CT analysis was also unable to give a true estimate of the number and size of femoral neck foramina. Only through painstaking macroscopic analysis of the nutrient foramina were we able to discern the true nature of the nutrient foramina within the femoral neck and their relationship to the retinacula of Weitbrecht.

Few studies have evaluated the nutrient foramina within the femoral neck (Lavigne et al. 2005; Sevitt and Thompson 1965; Jung et al. 1965; Tucker 1949). In a study by Lavigne et al. (2005), most (77 %) of the vascular foramina were located on anterolateral regions of the femoral neck, similar to our findings. Lavigne et al. (2005) also reported that vascular foramina were completely missing from the anterior neck in 71 % of the femora. The paucity of vascular foramina identified anteriorly and medially in our study has also been observed in other studies (Sevitt and Thompson 1965; Tucker 1949; Howe et al. 1950; Chung 1976; Ogden 1974), and has been explained by the regression of these vessels as the neck grows (Ogden 1974). Since many vascular foramina were observed in proximity to the anterolateral neck region, care should be taken when approaching this area during surgery.

In addition, this overall asymmetric distribution in nutrient foraminal location suggests that individual differences are clinically significant. In cases with relatively few nutrient foramina concentrated near the articular cartilage of the femoral head, even mildly displaced fractures, such as a valgus impacted femoral neck fracture, can affect the blood supply to the femoral head because of possible fatal entrapment of the blood vessels by the femoral head. In contrast, in cases with many nutrient foramina, although the fracture may damage some blood vessels, the residual intact blood vessels may still be able to provide the required blood supply to the femoral head.

Similar findings were noted with regards to nutrient foraminal shape. Some were round or oval and burrowed directly into the bone, while others appeared as bony grooves within the femoral neck. In the latter situation, the blood vessels may be easily broken during fracture due to a lack of surrounding bony support.

Individual variation in the size of femoral neck foramina has been reported (Tucker 1949). A study by Tucker (1949) reported retinacular artery diameters ranging from 0.10 to 1.55 mm in adults, with a mean diameter of 0.84 mm for the posterolateral retinacular vessels. We also found significant variation in foraminal size, in line with previous reports (Tucker 1949).

In summary, evaluation of the blood supply to the femoral head and neck has shown that individual differences exist and that such differences will affect the prognosis of patients with fractures. The results of this study have suggested that current imaging technology cannot fully reflect the extent of damage to the blood supply of the femoral neck after fracture. Future technology that would provide better screening of the blood supply after fracture could reduce complications, individualize treatment options (internal fixation vs. arthroplasty), and improve the success rate. Such technology may take the form of higher-resolution CT, or possibly micro-CT (Cooper et al. 2003).

Our study had several limitations. The sample size was relatively small and sampling error may have affected the results. In addition, no clinical data regarding the specimens were available for analysis. Although foramen number has not been linked to age, multivariate logistic analysis revealed a significant interaction between the effects of gender and sidedness on foramen number (*p* < 0.01) in a study of nutrient foramina within the human femoral diaphysis (Bridgeman and Brookes 1996). Although we hypothesized that there may be significant individual differences in the size and number of the nutrient foramina, and our results have also verified this hypothesis as reasonable, we have not found any related papers and this is a limitation of the study. In addition, we did not study the relationship between the length of the femoral bones and number/size of foramina within the femoral neck. Few studies have been published concerning the length of the femoral bone and its relationship to the number/size of foramina within the femoral neck. However, according to several studies (Murlimanju et al. 2011a, b, c; Kizilkanat et al. 2007), the distribution of the nutrient foramina within the femoral shaft is not related to the length of the femoral shaft. Larger prospective studies are needed, therefore, to provide clinical data regarding gender and sidedness and also to explore the effect of femoral shaft length on the number of nutrient foramina within the femoral neck. Future follow-up studies are also needed to further investigate the likely correlation between severity of injury to the retinacula and subsequent development of AVN.

Our ultimate goal is to be able to objectively evaluate the damage to the nutrient foramina in the femoral neck before selecting a surgical plan for each patient with a femoral neck fracture. This was based on our clinical observation that not all cases with seriously displaced femoral neck fractures developed AVN of the femoral head, while some cases with non-displaced femoral neck fractures developed AVN after internal fixations. When performing hemiarthroplasty or total hip replacement after femoral neck fractures, we also observed that the state of the blood supply to the femoral head differed significantly among cases with seriously displaced femoral neck fractures. In some cases there was a rich blood supply to the femoral head, while in others there was no blood supply at all to the femoral head, and the factors determining the extent of the blood supply included the richness of the nutrient foramina and the severity of injury to the retinacula of Weitbrecht. Unfortunately, we had already decided to perform joint replacement and removed the femoral head in these cases. Thus, we do not know what the ultimate prognosis of the patients with seriously displaced femoral neck fractures (who also had a rich blood supply) would have been if we had not performed hip joint replacement in these cases. Meanwhile, we also could not observe the distribution of the nutrient foramina in patients who underwent internal fixation. Therefore, we tried to use CT reconstruction to observe the states of nutrient foramina in the femoral neck, with a view to selecting an appropriate surgical procedure (internal fixation or joint replacement) by studying the distribution of the nutrient foramina or the severity of injury before surgery. Unfortunately, current CT scanners used in clinical practice (including some high-quality CT scanners) cannot meet our requirements in this regard. Although our observations have verified our hypothesis, more precise CT scanners are still needed to incorporate our technique into day-to-day clinical practice. We believe that this goal will be realized with future advancements in technology.

In conclusion, existing theory cannot explain why a nondisplaced femoral neck fracture can lead to AVN. It may be explained by the number and distribution of nutrient foramina within the femoral neck. We found that the foraminal distribution parallels the retinacula of Weitbrecht, a structure that provides support and protection for blood vessels entering the femoral head. In a condition in which there are few nutrient foramina, even if the fracture itself is nondisplaced, the damage to the nutrient foramina may cause osteonecrosis and arthroplasty is the best option. If there are many nutrient foramina, even in a displaced fracture, the nutrient foramina that have not been damaged can still provide sufficient blood supply to the femoral head, and internal fixation becomes an option.
